# Mesenchymal stem cells in the treatment of severe COVID-19

**DOI:** 10.1186/s41231-021-00095-0

**Published:** 2021-08-09

**Authors:** Santosh Kesari, Gregory C. Kasper, Lev Verkh, Terese C. Hammond, Marla L. Matal, Jay W. Hammerling, Nikolai Tankovich, Adrianus P. Lim, Kevin H. Zhao, Tiffany Juarez, Roberta E. Redfern, Jaya M. Gill, Natsuko Nomura, Audrey Hiemer, Annie Heng, Jessica Shoemaker

**Affiliations:** 1 Pacific Neuroscience Institute and Providence Saint John’s Health Center, Santa Monica, USA; 2 ProMedica Toledo Hospital, 2142 North Cove Blvd, Toledo, OH 43606 USA; 3 Stemedica Cell Technologies, Inc., San Diego, USA; 4 Providence Saint John’s Health Center, Santa Monica, USA; 5 Pacific Neuroscience Institute, Santa Monica, USA

To the Editor:

Researchers have hypothesized the utility of Mesenchymal Stem Cells (MSCs) in treating COVID-19. The severe form of the disease is due to an inflammatory cytokine storm, previously characterized in similar illnesses and possibly responsible for the development of Acute Respiratory Distress Syndrome (ARDS). MSCs have immunomodulatory properties, secreting anti-inflammatory cytokines, inhibiting monocyte differentiation, and regulating the function and proliferation of immune cells [1].

MSCs are used in the treatment of a range of immune-mediated inflammatory diseases such as lupus erythematous and graft versus host disease [2, 3]. Moreover, MSCs reduced mortality in H5N1 influenza and resulted in improved survival in those with H7N9 influenza strain who developed ARDS [1]. MSCs have activity in the treatment of ARDS, pneumonia, and sepsis, which are among the leading causes of death in COVID-19 cases [4]. Treatment strategies of ARDS is of particular importance in those with COVID-19 due to the potential long-term effects on pulmonary function, as pulmonary fibrosis can continue to develop even after the acute phase of the disease [5]. Despite the potential of MSCs for treating COVID-19, little patient data has been available thus far.

Here, we report a prospective case series of patients with laboratory confirmed COVID-19 treated with allogenic bone marrow-derived, ischemic tolerant hypoxic MSCs (Stemedica Cell Technology, San Diego, CA USA) at two hospitals in the United States. The data represents nine patients treated under emergency use IND and five treated under an expanded use IND at ProMedica Toledo Hospital (Toledo, OH USA) and Providence Saint John’s Health Center (Santa Monica, CA USA). All patients were categorized as severe or critically severe on admission, as previously defined [6]. Patient populations were somewhat dissimilar as shown in Table 1. Those treated under the expanded use were more severely ill, more often failed other potential COVID-19 treatments, and were infused with MSCs later in their clinical course. All expanded use and five emergency use cases were treated with 50 × 10^6^ cells twice, on average 5 days apart. Four emergency use patients were given a single bolus of 100 × 10^6^ cells, one of whom received approximately 64 M cells. Cells were delivered as an infusion in a central or peripheral line in an upper extremity as a constant rate of 2 M cells/minute. Dose per kg of body weight varied from 0.42 × 10^6^ cells/kg to 1.5 × 10^6^ cells/kg, 0.69 × 10^6^ cells/kg on average. Patients were tested for allergic skin reactions with subcutaneous microdose prior to infusion.

**Table 1:** Baseline, treatment, and outcome characteristics of patients treated with Mesenchymal Stem Cells (MSCs) under emergency use and expanded use indications

	All patients (*n* = 14)	Emergency use (*n* = 9)	Expanded use (*n* = 5)
Median age – years (range)	58.5 (30 – 84)	64 (56 – 84)	38.0 (30 – 59)
Female sex – n (%)	6 (42.9)	4 (44.4)	2 (40)
Comorbid conditions			
Cardiovascular disease – n (%)	1 (7.1)	1 (11.1)	0 (0)
COPD – n (%)	4 (28.6)	4 (44.4)	0 (0)
Hypertension – n (%)	7 (50)	7 (77.8)	0 (0)
Obesity – n (%)	8 (57.1)	3 (33.3)	5 (100)
Other pulmonary disease – n (%)	6 (42.9)	6 (66.6)	0 (0)
Type II diabetes – n (%)	3 (21.4)	2 (22.2)	1 (20)
COVID-19 Severity on admission			
Severe – n (%)	5 (35.7)	5 (55.6)	0 (0)
Critically severe – n (%)	9 (64.3)	4 (44.4)	5 (100)
Days of admission prior to treatment with MSCs – median (range)	10 (2 – 77)	4 (2 – 12)	41 (13 – 77)
Days between MSC treatments – median (range) ^a^	5 (2 – 8)	3 (2 – 3)	7 (6 – 8)
Failed or concurrent therapies			
Antibiotics	14 (100)	9 (100)	5 (100)
Antiviral	5 (35.7)	1 (11.1)	4 (80)
Convalescent serum	6 (42.9)	1 (11.1)	5 (100)
Hydroxychloroquine	8 (57.1)	5 (55.6)	3 (60)
Interleukin-6 inhibitor	6 (42.9)	1 (11.1)	5 (100)
Steroids	10 (71.4)	8 (88.9)	2 (40)
Oxygen therapy required			
Mechanical ventilation	10 (71.4)	5 (55.6)	5 (100)
ECMO	5 (35.7)	0 (0)	5 (100)
Pneumonia	13 (92.9)	8 (88.9)	5 (100)
Concurrent bacterial pneumonia	7 (50)	2 (22.2)	5 (100)
ARDS	9 (64.3)	4 (44.4)	5 (100)
LOS post-treatment	15 (2 – 50)	13 (2 – 50)	17 (15 – 25)
Total length of stay days ^b^	31.5 (5 – 98)	17 (5 – 63)	71 (45 – 98)
Survival – post treatment with MSCs			
7 days	13 (92.9)	8 (88.9)	5 (100)
14 days	12 (85.7)	7 (77.8)	5 (100)
21 days	12 (85.7)	7 (77.8)	5 (100)
30 days	9 (64.3)	7 (77.8)	2 (40)

^a^ 3 of 8 Emergency Use patients were treated with one bolus of 100 × 10^6^ cells

^b^ includes 2 patients admitted at time of writing

Following treatment with MSCs, four of five mechanically ventilated emergency use cases were extubated, exhibiting marked improvement on radiographic examination (Fig. 1). Objective severity category of ARDS as evidenced by PaO_2_/FiO_2_ [7] improved in three emergency use cases and one expanded use case within 21 days post-treatment. Laboratory results suggest that inflammatory markers were modulated by treatment with MSCs however were not significant in this small sample size. Overall survival was favorable; 92.9% of all cases survived 7 days post-treatment and 64.3% were alive at 30 days. Cause of death for both patients treated under the emergency use IND was cardiopulmonary failure; COVID-19-related ARDS was cited as the cause in all three patients who expired in the expanded use group. Importantly, no adverse events attributable to the MSC infusion were observed, suggesting the treatment is safe in this disease. At the time of writing, seven patients have been discharged. Five of the seven patients discharged from the hospital reported that their subjective overall function had returned to, or nearly to, baseline on follow up. One remains admitted to a long-term care facility; two others continue to be treated in the ICU.

**Fig. 1:**
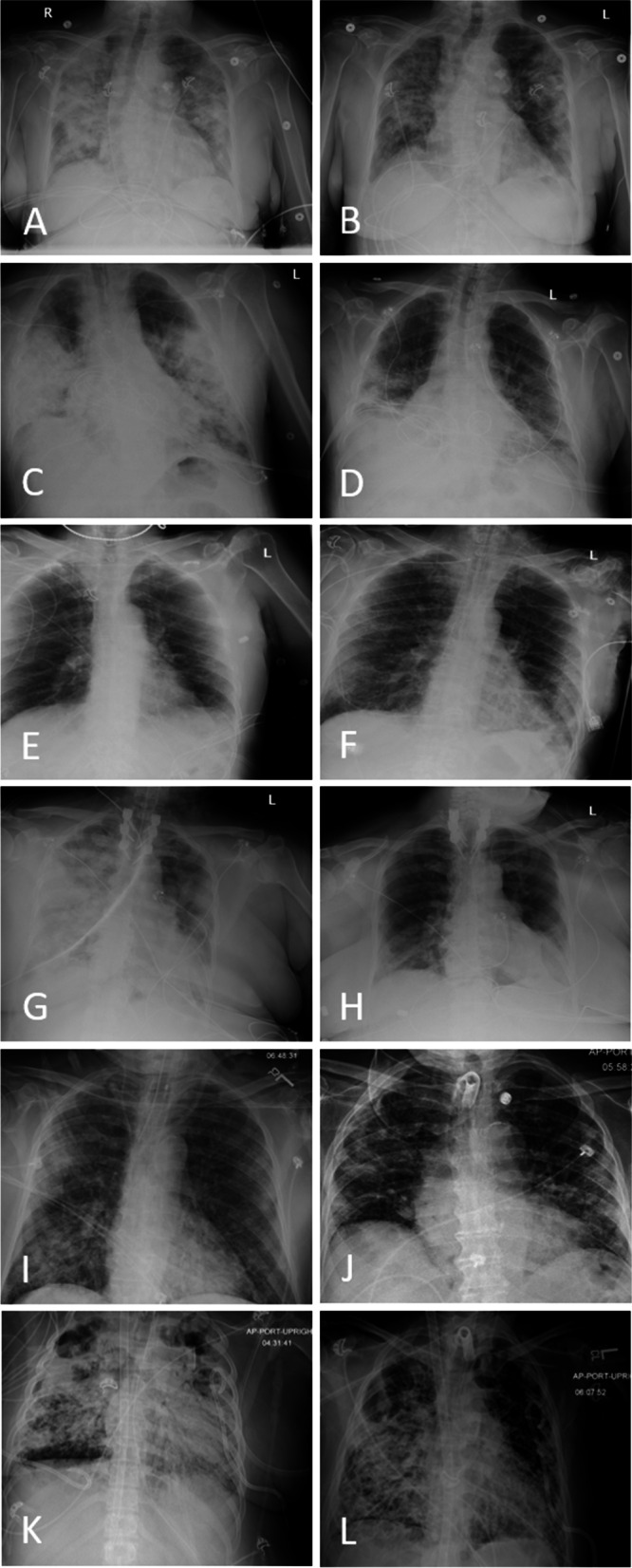
Chest radiographs of patient prior to and following treatment with Mesenchymal Stem Cells (MSCs). Seventy-nine year old female, with MSC treatment 2 days post-admission; chest radiograph day 0 showing bilateral infiltrates (**A**) and 12 days post-treatment (**B**). Sixty-four year old critically severe male, radiograph on day of ventilation and MSC treatment (**C**). Marked improvement of infiltrates and extubation 8 days following initial MSC infusion (**D**). 73 year old critically severe male treated with MSCs 12 days after initial admission. Radiograph the day prior to MSC treatment (**E**) indicates pulmonary infiltrates, which improved by day 9 following MSC infusion (**F**). Sixty-one year old female categorized as critically severe COVID-19 on admission, treated with MSCs 4 days following admission. Chest x-rays one day prior to MSC infusions indicates severe pulmonary infection (**G**) which substantially improved by day 11 post-treatment (**H**). Eighty-four year old male treated with MSCs 11 days after admission. Pre-treatment radiograph (**I**) on day 11 shows mild worsening of patchy airspace opacities bilaterally. There was slow improvement over time and infiltrates reduced on day 22 following MSC treatment (**J**). 30 year old female treated with MSCs 57 days after admission. Pre-treatment radiograph (**K**) on day 56 shows stable severe diffuse multifocal pulmonary opacities and focal lucencies within both upper lung zones which may represent loculated pleural air or parenchymal cavitation. Infiltrates reduced on day 4 following MSC treatment (**L**)

While this was not a clinical trial designed and powered for comparisons, our data suggest that those who received MSCs earlier in their clinical course of disease may have appreciated more benefit; it is possible that earlier treatment may help prevent the cytokine storm, rather than attempting to reverse it. While our data must be interpreted with caution given the limitations, it may provide some guidance for the design of future trials using MSCs to battle COVID-19.

## Data Availability

All data generated or analyzed during this study are included in this published article.
