# Correction to: Mesenchymal stem cells in the treatment of severe COVID-19

**DOI:** 10.1186/s41231-021-00098-x

**Published:** 2021-08-23

**Authors:** Santosh Kesari, Gregory C. Kasper, Lev Verkh, Terese C. Hammond, Marla L. Matal, Jay W. Hammerling, Nikolai Tankovich, Adrianus P. Lim, Kevin H. Zhao, Tiffany Juarez, Roberta E. Redfern, Jaya M. Gill, Natsuko Nomura, Audrey Hiemer, Annie Heng, Jessica Shoemaker

**Affiliations:** 1 Pacific Neuroscience Institute and Providence Saint John’s Health Center, Santa Monica, USA; 2 ProMedica Toledo Hospital, 2142 North Cove Blvd, Toledo, OH 43606 USA; 3 Stemedica Cell Technologies, Inc., San Diego, USA; 4 Providence Saint John’s Health Center, Santa Monica, USA; 5 Pacific Neuroscience Institute, Santa Monica, USA

## Background

**Correction to: Transl Med Commun 6, 16 (2021)**

**https://doi.org/10.1186/s41231-021-00095-0**

Following publication of the original article [1], the authors reported an error in the list of affiliations and requested to remove affiliation 2 “University of California San Diego Health System, Translational Neurosciences and Neurotherapeutics, Santa Monica, CA 90404, USA.”

The original article [1] has been updated.
